# Examining the Effectiveness of a Group Occupational Therapy Program During the Early Stage of Psychiatric Hospitalization in Japan: A Randomized Controlled Trial

**DOI:** 10.7759/cureus.85496

**Published:** 2025-06-07

**Authors:** Takeshi Sasaki, Atsuko Tanimura

**Affiliations:** 1 Department of Occupational Therapy, Ibaraki Prefectural University of Health Sciences, Ami, JPN; 2 Department of Occupational Therapy, Graduate School of Human Health Science, Tokyo Metropolitan University, Tokyo, JPN

**Keywords:** in-patient setting, mental health disorders, occupational participation, occupational therapy program, recovery

## Abstract

Background: In mental health occupational therapy, occupation-focused practice is recognized as crucial for supporting clients’ quality of life and facilitating their recovery. However, evidence supporting the effectiveness of group-based, occupation-focused interventions for individuals with mental disorders during the early stages of hospitalization remains limited.

Objective: This study aimed to develop and evaluate the effectiveness of a group occupational therapy program designed to promote occupational participation among individuals with mental disorders during the early stages of hospitalization.

Methods: A multi-center, open-label, randomized controlled trial was conducted. Participants were block-randomized in a 1:1 ratio by sex into an intervention group (novel program + standard occupational therapy) or a control group (standard occupational therapy only). The developed program was designed to identify clients’ occupations, clarify challenges in daily life, enhance motivation for occupational participation, and explore strategies to address barriers to occupational performance. It was implemented once a week over four sessions. Outcomes included the Japanese version of the Intrinsic Motivation Inventory (primary outcome), the Occupational Self-Assessment Short Form, the Occupational Questionnaire (OQ), the General Self-Efficacy Scale, and the Life Assessment Scale for the Mentally Ill (LASMI), all assessed pre- and post-intervention. Program evaluation questionnaires and qualitative feedback were collected from the intervention group. Analyses used hierarchical Bayesian linear models with weakly informative priors and the NUTS algorithm to estimate time-by-group interaction effects.

Results: A total of 13 participants were analyzed (intervention group: 10, control group: 3). Posterior estimates indicated meaningful interaction effects, suggesting that the intervention group showed greater improvements in interpersonal relationships and work on the LASMI, as well as in interest in occupation on the OQ, compared to the control group. Additionally, a potential positive effect was observed for the daily living subscale of the LASMI. Qualitative feedback highlighted participants’ awareness of meaningful occupations, reactivated motivation, and transformative changes in occupational participation and interpersonal relationships.

Conclusion: The study’s findings suggest that occupation-focused group interventions may enhance motivation, specifically, interest in meaningful occupations related to occupational participation and improve community living functions during early psychiatric hospitalization. The results support the clinical value of such programs as recovery-oriented practices in acute mental health care. Further large-scale studies are required to verify reproducibility, given the small sample size.

## Introduction

In mental health practice, occupation-focused practice plays a vital role in supporting clients’ quality of life and facilitating recovery. Specifically, recovery-oriented practice emphasizes individuals’ hopes and their engagement in meaningful activities, aligning closely with core occupational therapy values, such as occupation-focused practice, client-centeredness, and the achievement of personally meaningful goals. Recovery-oriented practice has become a central paradigm in mental healthcare [1]. In acute psychiatric inpatient settings, engaging in occupations, building interpersonal relationships, and experiencing personal growth from the early stages of hospitalization can serve as crucial means of promoting recovery. It has been demonstrated that interpersonal connections in sub-acute settings contribute significantly to recovery [2], while a systematic review reported that occupation-based group activities lead to positive outcomes, including social connectedness, a sense of achievement, and peer learning [3]. These experiences through occupation are effective in promoting both personal and clinical recovery and hold considerable value in acute psychiatric care. Participation in meaningful occupations, such as work and leisure, has been shown to contribute positively to personal recovery [4], suggesting the need for occupational therapists to provide occupation-focused interventions to support recovery.

However, there are a limited number of studies that have adopted occupational performance and participation as outcome measures during the acute phase or early stages of psychiatric hospitalization, which has been reported as a barrier to systematic implementation of occupation-focused practices in clinical settings [5]. Previous studies have reported that occupation-focused and recovery-oriented practices in acute care settings are often constrained by hospital environments and professional attitudes. For example, it has been noted that acute care environments frequently restrict opportunities for engagement in meaningful occupations, and mental health professionals may be reluctant to embrace recovery-oriented approaches [6,7]. Although group programs with an occupational focus have been developed and demonstrated effectiveness among older adults and community-dwelling individuals with mental disorders in Japan [8,9], their application and validation in early-stage inpatient settings remain insufficient. Thus, research on occupational engagement in acute psychiatric wards is limited. The clarification of occupation-focused practice and its effects in inpatient settings has also been identified as an important area for future research [10,11].

Therefore, the present study aims to develop an occupation-focused group occupational therapy program designed to promote occupational participation for individuals in the early stages of psychiatric hospitalization and to examine the effects of the program on occupational participation, motivation, and community living functions.

## Materials and methods

Study design

This study was a multi-center, unblinded, randomized controlled trial conducted in psychiatric hospitals, with two participating private hospitals located in the Tokyo metropolitan area. The objective was to evaluate the effectiveness of a group occupational therapy program aimed at promoting occupational participation among inpatients with mental disorders during the early stages of hospitalization. Participants were randomly assigned in a 1:1 ratio to either the intervention group, which received the program developed by the researchers, or the control group, which received only standard occupational therapy. Randomization was performed using block randomization stratified by sex following the completion of baseline assessments. A random number table was used to determine group allocation. Post-intervention assessments were conducted for all participants in the intervention group following program completion. For the control group, post-assessments were scheduled to either coincide with the intervention group’s post-assessment timing or occur approximately one month after the baseline assessment. The study period spanned from December 2023 to December 2024.

This study received approval from the Research Ethics Committee of Tokyo Metropolitan University (Approval Number: 22062). Additionally, the study protocol was registered with the University Hospital Medical Information Network Clinical Trials Registry (UMINCTR: UMIN000057271).

Participants

Recruitment notices were posted in the wards to invite participation in the study. Patients who expressed interest were screened for eligibility, and those who met the inclusion criteria were referred to the research team. The inclusion criteria were as follows: (i) currently hospitalized in a psychiatric facility for less than three months and (ii) aged between 18 and 65 years without a comorbid diagnosis of dementia. Additionally, participants were required to have the capacity to understand and respond to the assessment items and to provide informed consent for participation in the study. Eligibility was assessed by an attending occupational therapist and psychiatrist. Informed consent was obtained from all participants prior to their inclusion in the study. Exclusion criteria included cases where, due to physical or mental health conditions, the clinician judged that the individual would have difficulty participating in a 90-120-minute program session. This study did not use conventional sample-size estimation based on statistical power calculations. Instead, considering the characteristics of Bayesian statistics, the reliability of the parameter estimates was evaluated based on Markov Chain Monte Carlo (MCMC) samples obtained using the No-U-Turn Sampler (NUTS). Specifically, effective sample size (ESS) indicators, Bulk_ESS and Tail_ESS, were calculated to confirm that the sample size was sufficient for reliable estimation of intervention effects. Bulk_ESS and Tail_ESS represent the stability of estimation in the central and tail regions of the distribution, respectively, with values of 100 or more per chain (i.e., ≥ 400 in total) considered the threshold for reliability [12]. In this study, all estimations met or exceeded these thresholds, indicating acceptable reliability.

Intervention

In addition to standard occupational therapy, the intervention group received a structured, group-based occupational therapy program developed by the first author, which was based on four themes identified through semi-structured focus group interviews: (i) identifying the client’s occupations, (ii) enhancing motivation for occupational participation, (iii) providing opportunities for occupational experiences that facilitate change, and (iv) addressing difficulties in occupational participation arising from mental disorders.

The purpose of the program was to help participants identify challenges in their daily lives, strengthen their motivation to engage in various occupations, and explore strategies to address obstacles to occupational participation. The program consisted of four sessions that combined educational components on the relationship between occupation and health, grounded in the Model of Human Occupation (MOHO) [13]. The MOHO is a widely used occupational therapy model that explains occupational participation through the interaction of volition, habituation, performance capacity, and the environment, making it particularly relevant for understanding and supporting recovery in mental health settings. In addition, it addressed the relationship between the concepts of personal recovery and health, incorporating insights identified in previous research. Additionally, the program included self-reflection and discussions about clients’ lives and occupations based on the educational content, as well as activities focused on planning identified occupations. The program was implemented weekly over the course of one month (Table 1). The intervention commenced within 90 days of hospital admission and was conducted in small groups (3-4 participants per group, four groups in total) within occupational therapy rooms.

**Table 1:** Overview of the program.

Session	Theme	Learning Content	Structured Worksheet and Discussion
1st	Occupation and Health I	What is health?	Reflect on pre-admission occupations
		Concept of health: Relationship between occupation and health	Choose one occupation to try
		Explore different types of occupations	
2nd	Occupation and Health II	Concept of health: Recovery	Reflect on the occupation tried
		Explore occupations through connection with others	Engage in peer interaction
			Re-set a goal for the next occupation to try
3rd	Occupation and Health III	Consider occupation from the perspective of enjoyment	Reflect on the occupation tried
			Complete an interest checklist
			Create a plan to implement desired occupations
4th	Reflection and Future Prospects	Review of previous sessions and learning contents	Reflect on the occupations tried
			Explore personally meaningful occupations and consider future engagement

Each session began with a brief introduction (approximately five minutes), presenting the session theme and objectives. This was followed by a booklet-based educational segment and a peer discussion (approximately 30 minutes), individual and group work using worksheets (approximately 30 minutes), and a reflective segment that included planning for occupational activities to be practiced until the next session (approximately 30 minutes). The educational materials, which were developed by the first author, included booklets summarizing the core content and worksheets designed for structured reflection and planning. The first author facilitated all sessions.

The control group received standard occupational therapy, which included support for activities of daily living (ADL), various therapeutic activities, and psychosocial interventions such as psychoeducation (e.g., education on symptom management, medication adherence, stress coping strategies, and relapse prevention).

Assessment

Assessments were conducted at two time points: before randomization (baseline) and after the intervention (post-intervention). All evaluations were performed by occupational therapists using standardized procedures. These therapists were not involved in delivering the intervention program and were independent of the first author. The occupational therapists were aware of the group assignments and followed consistent protocols for data collection across all participants.

Demographic and Clinical Information

Basic information was collected through a review of medical records and interviews with the participants’ occupational therapists. This information included age, sex, diagnosis, comorbidities, number of hospitalizations, date of admission, length of hospital stay, and the date occupational therapy began.

Outcome Measures

Five outcome measures were used to evaluate intervention effects. The primary outcome was the Japanese version of the Intrinsic Motivation Inventory (IMI-J). The secondary outcomes included the Occupational Self-Assessment Short Form (OSA-SF), the Occupational Questionnaire (OQ), the General Self-Efficacy Scale (GSES), and the Life Assessment Scale for the Mentally Ill (LASMI). All outcome measures were administered using validated Japanese versions.

IMI-J [14] is a 21-item self-report measure that assesses three subdomains: interest/enjoyment, perceived choice, and value/usefulness. Items are rated on a 7-point Likert scale, ranging from “not at all true” to “very true.” Higher total scores indicate a greater intrinsic motivation toward a specific task.

OSA-SF is a shortened version of the Occupational Self-Assessment (OSA) [15], designed to evaluate clients’ self-perception of occupational competence and value based on the MOHO. The OSA-SF was created by reducing the original 21 items of the OSA to 12 through Rasch analysis [16]. The authors have verified the reliability and validity of the OSA-SF in healthy adults [17]. The OSA-SF is a 12-item self-report scale designed to assess two dimensions: competence and value. The competence scale uses a 4-point rating from 1 (I have a lot of problems doing this) to 4 (I do this extremely well), yielding scores from 12 to 48. The value scale uses a 3-point rating from 1 (This is not so important to me) to 3 (This is more important to me), yielding scores from 12 to 36. Higher scores indicate greater perceived competence and value in occupational engagement [16]. OQ [18] is a self-report tool designed to capture a client’s volition and engagement in various occupations over the course of one day. Participants recorded their main activity every 30 minutes while awake, classifying each as work, ADL, recreation, or rest. They then rated each activity across three dimensions: personal causation (how well they performed it), value (how important it was), and interest (how enjoyable it was), using a 5-point scale. This approach facilitated the analysis of subjective perceptions of activities and patterns of time use. In this study, we calculated the average time spent per activity category, as well as the mean scores for personal causation, value, and interest. 

GSES [19] consists of 16 dichotomous (yes/no) items that assess general self-efficacy in everyday life. Total scores can range from 0 to 16, with higher scores indicating stronger self-efficacy. 

LASMI [20] is a social functioning scale that comprises five subcategories: (i) daily living, (ii) interpersonal relations, (iii) work, (iv) endurance and stability, and (v) self-recognition. Each item is rated on a 5-point scale (no problem = 0 to a serious problem = 4). Lower scores indicate a higher degree of independent living within the community. In this study, we used the three subscales that are considered to represent community living functions: (i) daily living, (ii) interpersonal relations, and (iii) work [21].

Assessment of Program Effectiveness

Questionnaire and qualitative feedback from participants: In the intervention group, a program evaluation questionnaire and open-ended feedback were administered one week after each session. The questionnaire aimed to assess participants’ perceptions of the session across the following six items, each rated on a 5-point Likert scale (1 = strongly disagree to 5 = strongly agree): 1. How clearly did you understand the program’s content? (understanding of the program content), 2. Were you able to participate actively in the program? (level of participation), 3. Did you gain new knowledge or experience through the program? (acquisition of new knowledge), 4. Do you think that the content you learned will be useful in your daily life? (practicality of the content), 5. Have you noticed any changes in your daily life after participating in the program? (change in daily life), 6. How satisfied were you with the overall program? (overall satisfaction). Additionally, an open-ended section was provided for participants to freely describe their impressions of the program. Qualitative responses were collected to supplement the quantitative findings by capturing participants’ subjective experiences and perceived changes during the intervention.

Statistical analysis

Given the limited number of participants, this study employed a hierarchical Bayesian model for statistical analysis. Hierarchical Bayesian models are known to enhance the stability of estimations, even with small sample sizes [22]. They facilitate the estimation of group-level effects while accounting for individual-level variability and enable information sharing across different levels of data, thereby providing more reliable results. The analysis involved constructing a hierarchical Bayesian linear model that included the interaction between time (pre- and post-intervention) and group (intervention and control), treating individual participants as random effects to account for inter-individual differences. The model was estimated using the NUTS algorithm, which is implemented in the Hamiltonian Monte Carlo (HMC) method. Four chains were run, each with 4,000 iterations, including 1,000 burn-in iterations. The brms package (ver. 2.22.0) and CmdStanr (ver. 2.36.0) were used for model implementation. Model convergence was assessed using the Gelman-Rubin diagnostic statistic (Rhat), with values below 1.01 confirming convergence. Posterior means and 95% credible intervals (CrI) were calculated for effect estimation. To determine whether an intervention effect existed, we examined the interaction estimates between time and group. In addition to assessing whether the 95% CrI included zero, we used the probability of direction (pd), which reflects the directionality of the posterior distribution. A pd of ≥ 95% was interpreted as “possibly existing an effect,” and a pd of ≥ 97% as “likely to have an effect” [23,24]. Given the absence of clear prior expectations from previous studies and the need to avoid arbitrary estimations while maintaining flexibility to accommodate a wide range of phenomena, we adopted weakly informative priors. These priors are broadly recommended when strong prior knowledge is absent, as they impose reasonable constraints without inducing numerical instability [25]. Unlike non-informative priors, weakly informative priors help mitigate instability while avoiding overspecification. The priors used in this study were based on the official Stan guidelines [26].

## Results

Participants

**Figure 1:**
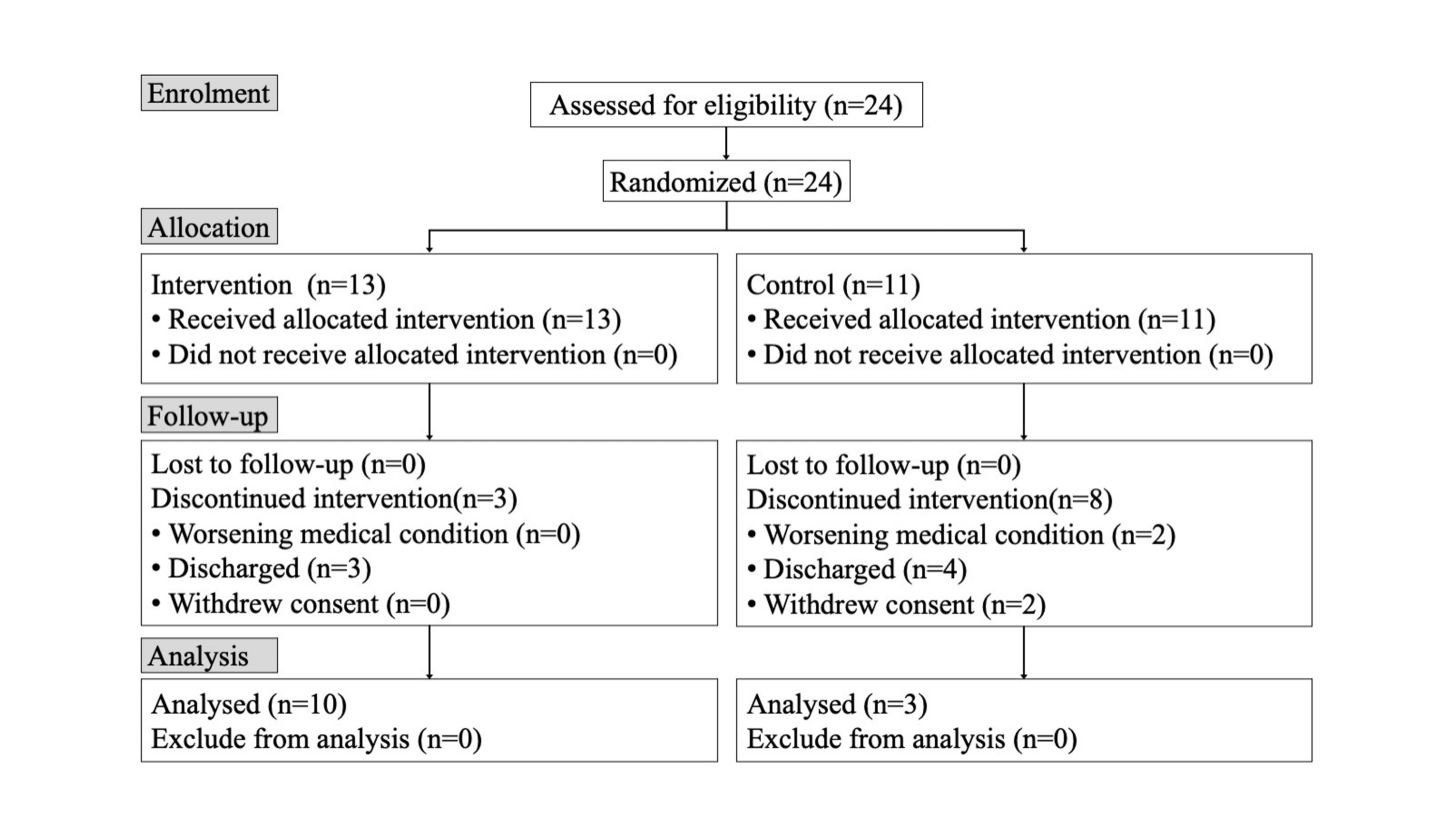
Study flowchart.

A total of 24 participants were enrolled in this study and randomly assigned to either the intervention group (n = 13) or the control group (n = 11) (Figure 1). Eleven participants dropped out, primarily due to a deterioration in their medical condition or discharge from the hospital. Ultimately, 13 participants (10 from the intervention group and three from the control group) were included in the final analysis. Demographic and clinical characteristics at baseline are presented using descriptive statistics (Table 2).

**Table 2:** Characteristics of participants.

Demographics	Intervention (n=10)	Control (n=3)
Age (years), mean (SD)	43.9 (12.46)	60.7 (2.08)
Sex, n (%)		
Female	6 (60.0)	3 (100.0)
Male	4 (40.0)	0 (0.0)
Diagnosis, n (%)		
Schizophrenia	5 (50.0)	3 (100.0)
Persistent depressive disorder	2 (20.0)	0 (0.0)
Adjustment disorder	1 (10.0)	0 (0.0)
Bipolar disorder	2 (20.0)	0 (0.0)
Number of hospital stays (times), mean (SD)	3.1 (2.77)	9.7 (7.64)
Total length of hospital stays (days), mean (SD)	30.7 (23.14)	45 (25.51)
Length of OT from hospitalization (days), mean (SD)	2.4 (2.37)	7.3 (6.51)

Estimation of intervention effects

For all outcome variables, the intervention effects were estimated using a hierarchical Bayesian model that incorporated the interaction between time (baseline and post) and group (intervention and control). A summary of the results is presented in Table 3. For all indicators, Rhat values were below 1.01, and both Bulk_ESS and Tail_ESS values exceeded 400, confirming MCMC convergence and estimation stability. Regarding the interaction estimates, three indicators showed 95% CrI that did not include zero, and the pd was ≥ 0.95. Specifically, among the LASMI subscales, interpersonal relations had an estimated value of -0.73 (95% CrI = -1.44 to -0.01, pd = 0.98), work was -0.76 (95% CrI = -1.36 to -0.12, pd = 0.99), and interest in the OQ was 0.63 (95% CrI = 0.01 to 1.22, pd = 0.98). When examining the mean and standard deviation by group for these indicators, the scores in the control group tended to worsen, while the intervention group showed improvement. Specifically, for interpersonal relations, the intervention group showed scores of pre = 1.50 (SD = 0.93) and post = 1.12 (SD = 0.83), while the control group exhibited pre = 1.31 (SD = 0.95) and post = 1.69 (SD = 1.39). For work, the intervention group was pre = 1.77 (SD = 1.21) and post = 1.36 (SD = 1.16), and the control group was pre = 2.27 (SD = 0.74) and post = 2.63 (SD = 0.83). For interest, the intervention group was pre = 3.86 (SD = 0.76), post = 4.17 (SD = 0.64), while the control group was pre = 3.91 (SD = 0.35), post = 3.58 (SD = 0.27). For the daily living subscale of the LASMI, the estimate was -0.38 (95% CrI = -0.80 to 0.05), which included zero in the CrI; however, the pd was relatively high at 0.96. For the remaining indicators, although the direction of the estimated effects appeared consistent, the 95% CrI included zero, and the pd was below 0.95. 

**Table 3:** Bayesian estimates for the interaction effects (time × group) and descriptive statistics (baseline, post) for each outcome variable. Estimate: Posterior mean, CrI: 95% credible interval, PD: probability of direction, Bulk_ESS and Tail_ESS: effective sample sizes for the posterior draws, Rhat: potential scale reduction factor (with Rhat < 1.01 indicating convergence), IMI-J: Japanese version of the Intrinsic Motivation Inventory, OSA-SF: Occupational Self-Assessment Short Form,  OQ: Occupational Questionnaire, GSES: General Self-Efficacy Scale, LASMI: Life Assessment Scale for Mentally Ill

Scale	Objective variable	Group	Baseline	Post	Interaction effect	PD	Rhat	Bulk_ESS	Tail_ESS
					Time × Group				
			Mean(SD)	Mean(SD)	Estimate [ 95%CrI ]				
IMI-J	Interest/enjoyment	Intervention	38.80 (5.14)	35.50 (8.02)	-1.34 [-4.50, 1.86]	0.80	<1.01	5391	5223
		Control	39.33 (6.66)	39.67 (10.97)					
	Perceived choice	Intervention	36.50 (8.83)	37.20 (6.78)	0.55 [-2.83, 3.82]	0.63	<1.01	5700	5307
		Control	33.00 (4.58)	32.33 (3.21)					
	Value/usefulness	Intervention	40.10 (6.54)	40.10 (6.51)	-0.31 [-3.24, 2.68]	0.59	<1.01	5339	5387
		Control	40.33 (7.09)	42.00 (8.89)					
	Total score	Intervention	115.40 (14.81)	112.80 (13.73)	-0.39 [-3.98, 3.23]	0.58	<1.01	4909	5048
		Control	112.67 (12.66)	114.00 (21.66)					
OSA-SF	Competence	Intervention	26.00 (9.07)	30.20 (9.15)	1.19 [-2.05, 4.38]	0.77	<1.01	5266	5228
		Control	22.33 (3.51)	26.33 (9.81)					
	Value	Intervention	24.20 (6.84)	25.10 (7.95)	-0.02 [-3.30, 3.43]	0.51	<1.01	5711	5231
		Control	23.33 (10.97)	28.00 (7.00)					
OQ	Personal causation	Intervention	4.18 (0.73)	4.28 (0.63)	0.25 [-0.53, 1.01]	0.75	<1.01	4947	4558
		Control	3.75 (0.22)	3.59 (0.60)					
	Values	Intervention	4.38 (0.59)	4.43 (0.41)	-0.14 [-0.84, 0.59]	0.67	<1.01	5337	5063
		Control	4.01 (0.37)	4.22 (0.35)					
	Interests	Intervention	3.86 (0.76)	4.17 (0.64)	0.63 [0.01, 1.22]	0.98	<1.01	4635	4487
		Control	3.91 (0.35)	3.58 (0.27)					
	Work (time)	Intervention	0.90 (1.51)	1.55 (1.69)	1.42 [-0.74, 3.41]	0.91	<1.01	4543	4465
		Control	2.00 (2.65)	0.17 (0.29)					
	Daily living task (time)	Intervention	5.70 (2.64)	7.70 (3.16)	0.94 [-1.69, 3.48]	0.77	<1.01	5324	5359
		Control	4.33 (1.04)	5.67 (1.15)					
	Recreation (time)	Intervention	5.85 (3.70)	4.35 (3.14)	-0.20 [-2.64, 2.23]	0.56	<1.01	4997	5306
		Control	4.33 (2.25)	2.83 (2.31)					
	rest (time)	Intervention	2.35 (3.42)	1.30 (1.77)	-1.11 [-3.62, 1.41]	0.80	<1.01	5357	5428
		Control	2.83 (0.76)	4.17 (3.01)					
GSES	Total score	Intervention	5.80 (3.91)	7.00 (4.67)	0.50 [-1.54, 2.54]	0.69	<1.01	4282	4890
		Control	2.67 (2.08)	3.67 (4.73)					
LASMI	Daily living	Intervention	1.49 (1.07)	1.23 (1.08)	-0.38 [-0.80, 0.05]	0.96	<1.01	4123	4102
		Control	1.89 (0.73)	2.00 (0.92)					
	Interpersonal relations	Intervention	1.50 (0.93)	1.12 (0.83)	-0.73 [-1.44, -0.01]	0.98	<1.01	4159	4669
		Control	1.31 (0.95)	1.69 (1.39)					
	Work	Intervention	1.77 (1.21)	1.36 (1.16)	-0.76 [-1.36, -0.12]	0.99	<1.01	4423	4367
		Control	2.27 (0.74)	2.63 (0.83)					

**Figure 2:**
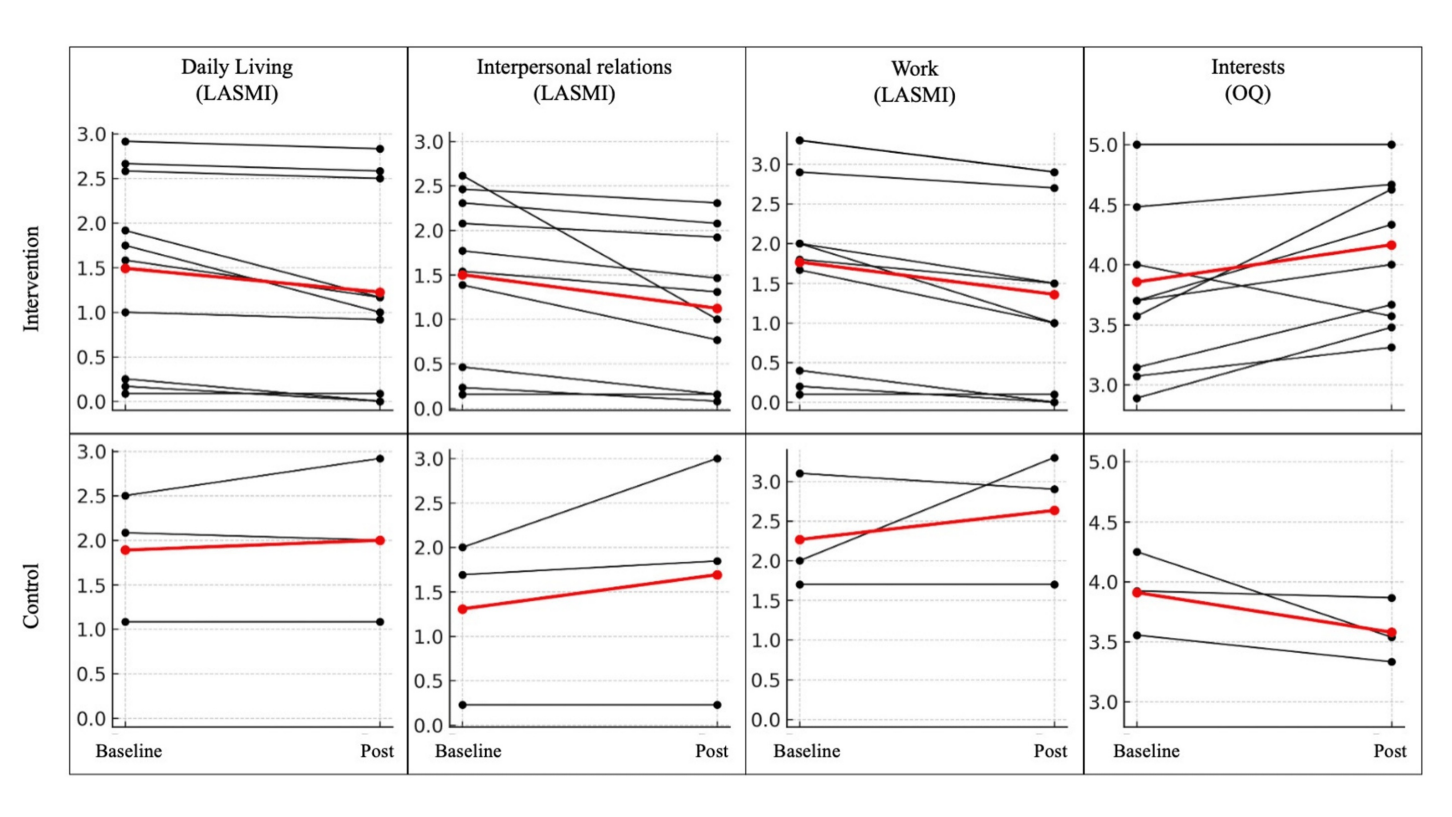
Pre- and post-intervention changes in outcome scores for each individual. Each black line represents the change in scores from baseline to post-intervention for an individual participant. The red line indicates the group mean at each time point. The figure displays four outcome domains that showed significant or suggestive interaction effects in the Bayesian analysis: Daily living, Interpersonal relations, Work (LASMI: Life Assessment Scale for the Mentally Ill), and Interests (OQ: Occupational Questionnaire).

As the control group was limited to individuals diagnosed with schizophrenia, Table 4 presents descriptive statistics for each of the relevant outcome measures restricted to this subsample. Additionally, to examine whether individual-level changes aligned with the overall trends, we visualized pre-post changes for each individual by group for the outcome measures that showed significant interaction effects (Figure 2). The graphs indicate that most participants in the intervention group showed improvements, while those in the control group tended to show deterioration, consistent with the overall trends.

**Table 4:** Descriptive statistics for outcome measures (schizophrenia subsample). Data are restricted to participants diagnosed with schizophrenia. OQ: Occupational Questionnaire; LASMI: Life Assessment Scale for the Mentally Ill.

Scale	Objective variable	Group	Baseline	Post
			Mean(SD)	Mean(SD)
OQ	Interests	Intervention	4.35 (0.69)	4.66 (0.41)
		Control	3.91 (0.35)	3.58 (0.27)
LASMI	Daily living	Intervention	1.75 (1.12)	1.50 (1.19)
		Control	1.89 (0.73)	2.00 (0.92)
	Interpersonal relations	Intervention	1.74 (1.01)	1.23 (0.88)
		Control	1.31 (0.95)	1.69 (1.39)
	Work	Intervention	2.02 (1.24)	1.74 (1.13)
		Control	2.27 (0.74)	2.63 (0.83)

Qualitative findings from participant feedback

In the intervention group, the post-program questionnaire results indicated high ratings across all items, including the comprehensibility of the content, overall satisfaction, and the acquisition of new knowledge (Table 5). Additionally, the free-text responses included comments related to participants’ understanding of occupation, recovery, and a sense of self through the program. Specific examples included statements such as, “It was the first time I considered about the quality of life through occupation. I found it very educational,” and “I reconsidered the definition of recovery.” Furthermore, comments concerning interpersonal relationships, self-disclosure, and family dynamics were also reported, including, “Since I don’t usually disclose much about myself, this was a very stimulating and meaningful experience,” and “I noticed changes in how I interact with my family-something I hadn’t been aware of before.” Other responses described concrete behavioral changes in daily life, such as, “I am now able to take a bath every day,” “I was able to identify things I want to do using the checklist,” and “I found an occupation I want to continue in the future” (Table 6).

**Table 5:** Summary of participant feedback on program sessions.

Item (Questionnaire)	1st	2nd	3rd	4th
	Mean(SD)	Mean(SD)	Mean(SD)	Mean(SD)
1. Understanding of the program content	4.70 (0.48)	4.00 (1.22)	4.44 (0.53)	4.38 (0.92)
2. Level of participation	4.60 (0.52)	4.22 (1.30)	4.56 (0.53)	4.38 (0.92)
3. Acquisition of new knowledge	4.60 (0.52)	4.44 (1.01)	4.33 (0.71)	4.50 (0.53)
4. Practicality of the content	5.00 (0.00)	4.56 (0.73)	4.56 (0.53)	4.63 (0.52)
5. Changes in daily life	4.30 (0.82)	3.89 (1.17)	3.89 (1.27)	4.63 (0.52)
6. Overall satisfaction	4.60 (0.52)	4.11 (0.93)	4.33 (0.71)	4.63 (0.52)

**Table 6:** Participant comments by session.

Session	Comments
1st	I hope to further develop my ability to apply what I’ve learned. I also realized how important it is to listen and understand.
	It was the first time I considered the quality of life through occupation. I found it very educational.
	I learned a lot.
	I am now able to take a bath every day
	I was able to clearly organize my thoughts about “occupation” in daily life.
	I learned that OT activities are a form of treatment, which was encouraging. It helped me become more engaged.
	I started to distinguish between activities of daily living and other types of occupations. Right now, I feel like I’m just resting in my daily life.
2nd	I felt a desire to improve and develop my personal skills and special abilities.
	Since I don’t usually disclose much about myself, this was a very stimulating and meaningful experience.
	I reconsidered the definition of “recovery.
	I realized that although I’m not good at interacting with others, those relationships are still very important.
	I noticed changes in how I interact with my family—something I hadn’t been aware of before.
3rd	The content was easy to understand.
	I discovered things I enjoy and have an interest in.
	Regarding my sleep issues, I now feel I need to experiment and carefully examine ways to improve.
	I was able to reconfirm my own interests. I realized again how much I enjoy hands-on activities.
	I’m very satisfied.
	My understanding of what makes me “myself” has deepened.
	I was able to identify things I want to do using the checklist. I like occupations that allow me to connect with others.
4th	It was a good opportunity to look at myself objectively, and I was able to better visualize my life after discharge.
	It was a session that made me reflect on the importance of human relationships.
	I gained useful insights for the future, thank you very much!
	I found an occupation I want to continue in the future. There are still many things I haven’t tried, so I’d like to explore and give them a go. I was able to discover things through the program that I wouldn’t have thought of on my own.

## Discussion

The impact of the program on occupational participation, community living function, and motivation

This study aimed to evaluate the effects of a group occupational therapy program designed to promote occupational engagement among individuals with mental disorders in the early stages of psychiatric hospitalization. The analysis revealed significant interaction effects in the intervention group for indicators of community living function (LASMI: interpersonal relations and work) and interest in occupations (OQ: interests), suggesting that these changes were attributable to the nature of the implemented program. Additionally, for the daily living subscale (LASMI), although the 95% Crl included zero, the pd suggested a potential effect of the intervention. Consistently, a similar trend was also observed in the descriptive statistics limited to participants with schizophrenia, supporting the findings of the primary analysis.

Improvements in community living functions may reflect how the program helped participants identify meaningful occupations, encouraged their engagement in daily life, and provided opportunities for experiential learning, thereby enhancing their functional skills. Moreover, group-based, occupation-focused interventions likely contributed to broader benefits in social interactions. Comments from participants, such as “I am now able to take a bath every day” and “I noticed changes in how I interact with my family-something I hadn’t been aware of before,” further support the improvement in daily life functioning. Notably, the direction of score changes for community living function outcomes differed between the intervention and control groups, indicating a clear effect of the program. This pattern was not only evident in the average scores but was also consistently observed in the individual trajectories of many participants, supporting the overall trend. The occupation-focused intervention, Balancing Everyday Life, demonstrated that reconstructing occupational balance through group work led to improved Global Assessment of Functioning scores, independent of symptom changes [27]. The present findings support the effectiveness of occupation-focused, group-based interventions for enhancing functional outcomes, even during the early stages of hospitalization. Unlike interventions that merely provided activities, this program supported participants in rediscovering “meaningful occupations” and incorporating them into their lives. It has been suggested that a stronger meaning attributed to occupation is associated with greater intervention effects [28]. Therefore, the improvements in interest and interpersonal functioning observed in this study may be attributed to the enhanced occupational meaning. 

Furthermore, although no interaction effects were observed for intrinsic motivation, including interest as measured by the IMI-J, or for self-efficacy as measured by the GSES, it is noteworthy that an increase in interest in overall daily life was reported. Unlike the IMI-J, which evaluates interest in specific tasks, the OQ assesses subjective perceptions across a broad spectrum of daily life occupations [14,18]. These findings suggest that, through the process of reflecting on their own occupations and daily activities, the program enhanced participants’ motivation toward their overall lives. This can be explained by the theory of the remotivation process in MOHO. According to this theory, volition develops through the stages of exploration, competency, and achievement. Exploration is defined as learning about one’s interests and values through self-selected trials of new occupations; competency involves developing new skills and enhancing self-efficacy; and achievement refers to the full development of skills and the habituation of occupations [29]. The present intervention likely facilitated the “exploration” stage, thereby enhancing interest and engagement in daily life and occupations. Participant feedback, such as “I discovered things I enjoy and have an interest in” and “I found an occupation I want to continue in the future. There are still many things I haven’t tried, so I’d like to explore and give them a go. I was able to discover things through the program that I wouldn’t have thought of on my own,” suggests that participants are experiencing changes in their awareness of their own occupations, as well as the formation of positive outlooks and hope for their future lives. However, the one-month duration may have been too brief for participants to progress to the competency stage or to experience significant increases in self-efficacy. Given these considerations, this program can be regarded as an initial intervention that encourages the exploration of occupations and their incorporation into daily life, ultimately leading to lifestyle changes and the enhancement of self-efficacy in the future. This is all grounded in the knowledge of occupation and health gained through the educational sessions. Following these interventions, it will likely be necessary to further strengthen motivation through support for continued occupational engagement and assistance in progressing to the competency stage.

Within the recovery-oriented perspective, which is central to the field of mental health, recovery is considered a gradual process of re-engagement through “doing.” Engagement in meaningful occupations is regarded as an important starting point [30]. In this program, participants were encouraged to explore occupations aligned with their interests and values and to incorporate these activities into their daily lives. This approach can be interpreted as directly supporting occupational participation in the early stages of recovery, thereby suggesting the effectiveness of recovery-oriented occupational therapy. In Japan, the effectiveness of such recovery- and occupation-focused interventions for community-dwelling people with mental disorders has been demonstrated [9]. However, there is a lack of equivalent evidence in inpatient settings, particularly regarding interventions in the early stages of hospitalization. The program in this study targeted individuals during the initial phases of inpatient psychiatric care and was grounded in an educational understanding of the relationship between occupation and health. By facilitating the rediscovery of meaningful occupations and providing opportunities for occupational engagement, the program was considered valuable in promoting lifestyle changes and fostering motivational transformation among participants.

Implications for occupational therapy practice

Based on the results of the participant questionnaire regarding the program, many participants responded “very satisfied” and “it was educational,” and high scores were observed across all indicators, including comprehension of the content, degree of participation, and overall satisfaction, confirming that the program was both acceptable and useful for the participants. However, although this study provided an occupational therapy program in a group format, the nature of targeting individuals with mental disorders within three months of hospitalization posed challenges. Some participants found it difficult to continue in the study due to fluctuations in their symptoms or the influence of treatment schedules. Considering these practical challenges, it is suggested that future implementations explore flexible formats that combine individual interventions. This approach may enhance responsiveness to a more diverse range of participants and improve feasibility. In particular, as this program is structured to promote self-understanding and the reconstruction of occupation, it possesses characteristics that would allow it to be sufficiently applicable, even if some sessions were modified to individual formats. Therefore, verifying the effectiveness of a combined group and individual format in future studies is expected to contribute to the development of a more practical and sustainable intervention model.

Study limitations and future directions

This study is significant in that it developed and evaluated a group occupational therapy program aimed at promoting occupational participation among psychiatric inpatients. To date, there has been a lack of evidence regarding the effectiveness of occupational therapy in the early stages of psychiatric hospitalization, and this study provides valuable insights that contribute to this field. A hierarchical Bayesian model was employed to estimate intervention effects while accounting for individual differences, allowing for stable estimation even with a small sample size. Furthermore, by integrating both quantitative and qualitative data, the study was able to comprehensively capture the effects of the intervention. Despite these strengths, this study has several limitations. First, the long-term effects of the intervention were not examined; therefore, it remains unclear whether the observed improvements in occupational participation and motivation will persist after discharge. A longitudinal study with a follow-up period is needed to assess the durability of the intervention’s effects. Second, the sample size was small, so future studies should include larger samples to enhance generalizability. In addition, while the control group was limited to individuals diagnosed with schizophrenia, the intervention group included participants with a variety of diagnoses. This imbalance in baseline characteristics between the groups may have influenced the comparison of intervention effects, and caution is warranted when interpreting the results. Third, while qualitative data were collected via participants’ free-text responses, the depth and scope of these responses were limited, which restricted the understanding of individual processes and the meaning of change. To gain deeper insights, future research should incorporate methods such as semi-structured interviews to explore participants’ subjective experiences in greater detail. Fourth, although the program was structured around standardized themes and content, the intervention effects may vary depending on the person delivering it. Future research should examine the reproducibility of the program when implemented by different occupational therapists.

## Conclusions

This study evaluated the effects of a group occupational therapy program aimed at promoting occupational participation among individuals in the early stages of psychiatric hospitalization. The results showed positive intervention effects in domains such as community living function (LASMI) and interest in occupations (OQ) when compared to a control group that received standard occupational therapy. The findings suggest that a group-based, occupation-focused program may facilitate the recovery of functional capacity and foster motivation. Additionally, qualitative feedback revealed shifts in participants’ perceptions and behaviors regarding occupations, confirming the significance of supporting occupational engagement during the initial recovery phase. In summary, this program demonstrates potential effectiveness and clinical applicability as an occupation-focused group intervention in acute psychiatric settings.

## Appendices

Questionnaire and qualitative feedback from participants

How clearly did you understand the program’s content?

-        Very clear

-        Clear

-        Neutral / Not sure

-        Not very clear

-        Not clear at all

Were you able to participate actively in the program?

-        Strongly agree

-        Somewhat agree

-        Neither agree nor disagree

-        Somewhat disagree

-        Strongly disagree

Did you gain new knowledge or experience through the program?

-        Strongly agree

-        Somewhat agree

-        Neither agree nor disagree

-        Somewhat disagree

-        Strongly disagree

Do you think that the content you learned will be useful in your daily life?

-        Strongly agree

-        Somewhat agree

-        Neither agree nor disagree

-        Somewhat disagree

-        Strongly disagree

Have you noticed any changes in your daily life after participating in the program?

-        Strongly agree

-        Somewhat agree

-        Neither agree nor disagree

-        Somewhat disagree

-        Strongly disagree

How satisfied were you with the overall program?

-        Very satisfied

-        Satisfied

-        Neutral / Not sure

-        Not very satisfied

-        Not satisfied at all

Please freely describe your impressions of the previous program
